# HDAC9 exacerbates endothelial injury in cerebral ischaemia/reperfusion injury

**DOI:** 10.1111/jcmm.12803

**Published:** 2016-02-10

**Authors:** Weichen Shi, Xinbing Wei, Ziying Wang, Huirong Han, Yi Fu, Jiang Liu, Yan Zhang, Jian Guo, Chuanqiao Dong, Di Zhou, Quan Zhou, Yuxin Chen, Fan Yi

**Affiliations:** ^1^ Department of PharmacologyShandong University School of MedicineJinanChina; ^2^ Department of Hepatobiliary SurgeryQilu Hospital of Shandong UniversityJinanChina

**Keywords:** histone deacetylase, ischaemic stroke, blood–brain barrier, autophagy, gene therapy

## Abstract

Histone deacetylase (HDAC) 9, a member of class II HDACs, regulates a wide variety of normal and abnormal physiological functions, which is usually expressed at high levels in the brain and skeletal muscle. Although studies have highlighted the importance of HDAC‐mediated epigenetic processes in the development of ischaemic stroke and very recent genome‐wide association studies have identified a variant in HDAC9 associated with large‐vessel ischemic stroke, the molecular events by which HDAC9 induces cerebral injury keep unclear. In this study, we found that HDAC9 was up‐regulated in the ischaemic cerebral hemisphere after cerebral ischaemia/reperfusion (I/R) injury in rats and *in vivo* gene silencing of HDAC9 by recombinated lentivirus infection in the brain reduced cerebral injury in experimental stroke. We further demonstrated that HDAC9 contributed to oxygen‐glucose deprivation‐induced brain microvessel endothelial cell dysfunction as demonstrated by the increased inflammatory responses, cellular apoptosis and endothelial cell permeability dysfunction accompanied by reduced expression of tight‐junction proteins. We further found that HDAC9 suppressed autophagy, which was associated with endothelial dysfunction. This study for the first time provides direct evidence that HDAC9 contributes to endothelial cell injury and demonstrates that HDAC9 is one of critical components of a signal transduction pathway that links cerebral injury to epigenetic modification in the brain.

## Introduction

Although the pathogenesis of ischaemic cerebral injury is multifactorial and strategies aimed to reduce oxidative stress, inflammation and intracellular calcium release have been considered for the treatment of stroke 1, the pre‐clinical protective agents targeting a specific pathway failed to demonstrate clinical efficacy. Thus, finding better therapeutic targets to prevent cerebral injury has been a great challenge in neurobiology. Recently, studies have highlighted the importance of epigenetic mechanisms such as histone modifications, DNA methylation, chromatin remodelling and non‐coding RNAs (ncRNAs) in the pathogenesis of stroke, which orchestrate almost every aspect of central nervous system (CNS) 2. Among them, histone deacetylases (HDACs)‐mediated epigenetic mechanisms play important roles in the homoeostasis of histone acetylation and gene transcription. There are four major classes of HDACs that are classified based on their structures and expression patterns 3. Class I (HDACs 1, 2, 3, and 8), class II (HDACs 4, 5, 6, 7, 9, and 10) and class IV (HDAC 11) are Zn^2+^‐dependent for enzymatic activity; whereas the class III sirtuins (SIRT1‐7) are NAD^+^‐dependent. Although HDAC inhibitors have neuroprotective properties in animal models for various neurological diseases including Alzheimer's disease and ischaemic stroke 4, 5, current HDAC inhibitors are mostly nonselective, and different HDACs serve very distinct functions. For instance, HDAC1 gain‐of‐function transgene exhibits potent protection against DNA damage and neurotoxicity 6 rather than the deterioration of cerebral injury 7, 8. Therefore, it is necessary to elucidate the functional role of individual HDACs in ischaemic stroke. Our previous studies have characterized the expression patterns of individual HDACs in rats after cerebral ischaemia/reperfusion (I/R) injury. We found that among Zn^2+^‐dependent HDACs, HDAC4 and HDAC5 were markedly decreased in ischaemic stroke and demonstrated that HDAC4 and HDAC5 protected cells from death through reducing HMGB1 expression and release 9. Interestingly, we have also observed that HDAC9 was significantly up‐regulated in the ischaemic brain 9. However, the role of HDAC9 in ischaemic stroke keeps unknown.

HDAC9, a member of class II HDACs, regulates a wide variety of normal and abnormal physiological functions, which is usually expressed at high levels in the brain and skeletal muscle. Although very recent genome‐wide association studies have identified a variant in HDAC9 associated with large‐vessel ischaemic stroke 10 and further demonstrated that HDAC9 genetic variant associated with ischaemic stroke increases risk *via* promoting carotid atherosclerosis 11, the molecular mechanisms of HDAC9 in ischaemic stroke are still largely unclear. In this study, we used the *in vivo* transient middle cerebral artery occlusion (MCAO) model and *in vitro* cell cultures *via* oxygen‐glucose deprivation (OGD) to investigate the role of HDAC9 in ischemic cerebral injury and found that HDAC9 promotes endothelial dysfunction in experimental ischaemic stroke.

## Materials and methods

### Animal models for transient focal cerebral ischaemia

Transient MCAO was performed in male Sprague–Dawley rats (250–280 g) as described 9, 12. In brief, anaesthesia was induced using 10% chloral hydrate (350 mg/kg). The left common and external carotid arteries were isolated and ligated. A nylon monofilament was introduced from the common carotid artery into the internal carotid artery until a resistance was encountered, thus blocking the origin of the middle cerebral artery. A successful occlusion was indicated by a decrease in the regional cerebral blood flow (rCBF) to <20% of the baseline by transcranial laser‐Doppler (Perimed, Jarfalla, Sweden) measurement in the area of cerebral cortex supplied by the MCA. After 2 hrs of MCAO, the suture was removed and reperfusion was confirmed by an immediate increase in rCBF. During and after the surgery, rectal temperature was controlled with a homoeothermic blanket and kept at 37°C until the complete recovery of the animal from the anaesthesia. After reperfusion at 24 or 48 hrs, the rats were anaesthetized and then killed. Overall, about 15% of animals died during surgery or after recovery from surgical anaesthesia and 12% percent of all animals used in this study were excluded because of insufficient CBF reduction. The percentage of excluded animals was similar across different groups. The number of mice used in this study for data collection and analysis was eight per group. All protocols were approved by Institutional Animal Care and Use Committee of Shandong University and conducted in accordance with the National Institutes of Health Guide for the Care and Use of Laboratory Animals.

### Lentivirus production and stereotaxic injection

Recombinant lentivirus vectors pGLV3/H1/GFPtPuro (pGLV3) harbouring a short‐hairpin RNA sequence targeting HDAC9 (pGLV3‐shRNA‐HDAC9) were produced by GenePharma (Shanghai, China). The pGLV3‐shRNA‐HDAC9 or their scramble (pGLV3‐null) was delivered to the rat cortex by means of intracortex injections as shown in Figure 2A and the procedure for intracortex lentivirus delivery was performed as described 13, 14. Briefly, in anaesthetized rats, two points for injection were selected. Point A 1.0–2.0‐mm anterior to the bregma and 3.0‐mm lateral to the midline, the injection depth was 3.0 mm; Point B 0.5–1.5‐mm posterior to the bregma and 3.5‐mm lateral to the midline, the injection depth was 3.5 mm. Both of the two points were in the left side. The speed of injection was 0.5 μl/min. The needle was carefully removed 10 min after the injection to avoid the reflux of the cerebrospinal fluid. As the needle was being slowly removed, 9 μl purified lentivirus (1 × 10^9^ TU/ml) or 9 μl scramble were injected. The dose of lentivirus was chosen based on our preliminary experiments and previous studies 15. Preliminary studies indicated that lentiviral‐mediated green fluorescent protein (GFP) expression in rat cortex was significantly increased after 1 week and no toxicity was observed in rats treated with lentiviral vectors.

### Infract volume and neurological function assessment

Stroke outcome was assessed at 48 hrs after reperfusion using cerebral infarct volume and a 4‐tiered neurological scoring system for cerebral injury assessment as described previously 16. Blood–brain barrier (BBB) permeability was estimated by Evan's blue leakage as described 17.

### Immunofluorescence

Immunofluorescent staining was performed as described 18. Images were obtained by confocal laser‐scanning microscopy using a LSM780 laser scanning confocal microscope (Zeiss, Oberkochen, Germany) equipped with a Plan‐Apochromat 63×/1.4 objective. Images were assembled in the Adobe Photoshop 7.0 software package.

### Cell culture and treatments

Primary cortical neuron, microglia and astrocytes were isolated and cultured as described 19. Primary brain microvessel endothelial cells (BMVECs) were prepared as described 20. The purity of neuron, microglia, astrocytes and BMVECs was evaluated by immunofluorescence staining using antibodies against NeuN (Cell Signaling Technology, Danvers, MA, USA), CD11b (BD Biosciences, San Diego, CA, USA), glial fibrillary acidic protein (GFAP; Invitrogen, Gaithersburg, MD, USA) and CD34 (BD Biosciences) respectively. Cells were subjected to the model of OGD (1‐hr of OGD followed by different reoxygenation duration time) as described 15. To study the function of HDAC9‐mediated autophagy on the endothelium, the BMVECs were pre‐treated with the autophagic activator rapamycin (10 μg/ml, R0395; Sigma‐Aldrich, St. Louis, MO, USA) for 2 hrs before OGD incubation.

### RNA interference and overexpression of HDAC9

Small interference RNA to HDAC9 (siRNA‐HDAC9) and its negative control were synthesized and constructed into pRNAT‐U6.1/Neo to get shRNA‐HDAC9 by Biomics Biotechnologies Co., Ltd. (Nantong, Jiangsu, China). The DNA target sequence for shRNA‐HDAC9 is 5′‐GCCAGUAUGGAAGUGGCAUTT‐3′. In these experiments, shRNA‐HDAC9 was transfected into BMVECs by Lipofectamine 2000 (Invitrogen) according to the manufacturer's instruction. For overexpression of HDAC9, BMVEC cells were transfected with pGV230‐plasmid (overexpression of HDAC9) or its negative control produced by GeneChem (Shanghai, China) *via* Lipofectamine 2000 according to the manufacturer's instruction.

### RNA extraction and real time RT‐PCR

Total RNA was isolated from the brain or cells, mRNA levels were analysed by real‐time RT‐PCR using a Bio‐Rad iCycler system (Bio‐Rad, Hercules, CA, USA) 19. The specific primers for target gene and housekeeping gene sequences were shown in Table S1.

### Western blot analysis

Total cellular lysates preparation and Western blot analysis were performed as described previously 21. Antibodies used in this study were summarized in Table S2. To document the loading controls, the membrane was re‐probed with a primary antibody against housekeeping protein β‐actin.

### Statistics

Data are expressed as means ± S.E. The significance of the differences in mean values between and within multiple groups was examined by one‐way anova followed by Duncan's multiple range test. *P* < 0.05 was considered as statistically significant.

## Results

### HDAC9 was significantly up‐regulated after cerebral I/R injury in rats

**Figure 1:**
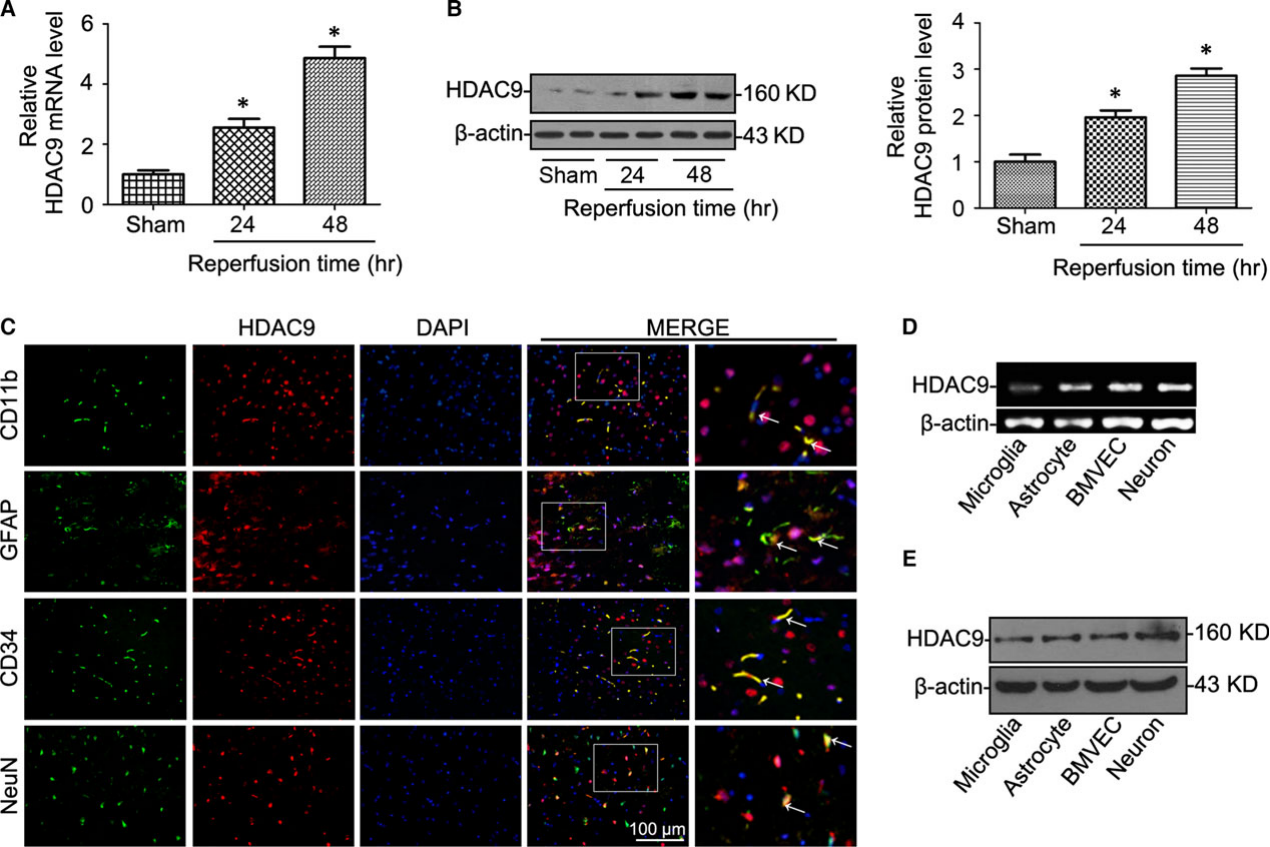
HDAC9 expression was significantly increased after cerebral ischaemia reperfusion (I/R). (**A**) Relative mRNA levels of HDAC9 by real‐time RT‐PCR analysis in the ischemic cerebral hemisphere of rats at different time‐points after reperfusion. (**B**) Western blot analysis of HDAC9 protein levels in the ischaemic cerebral hemisphere of rats at different time‐points after reperfusion. (**C**) Confocal immunofluorescent analysis showing that HDAC9 was expressed in different cell types including neuron, astrocytes, microglia and BMVECs in the brain of rats. We identified neurons by staining for the neuronal nuclear marker NeuN, astrocytes by staining for GFAP, activated microglia/macrophages by staining for CD11b and BMVECs by staining for CD34. (**D**) HDAC9 mRNA detection by RT‐PCR from primary cortical neuron, microglia, astrocytes and brain microvessel endothelial cells (BMVECs). (**E**) Representative Western blot gel documents showing HDAC9 protein levels in primary cortical neuron, microglia, astrocytes and BMVECs. *P < 0.05 *versus* sham‐operated rats (*n* = 8).

Cerebral injury after MCAO in rats was confirmed by triphenyltetrazolium chloride (TTC) staining and neurological deficit score. Real time RT‐PCR (Fig. 1A) and Western blot (Fig. 1B) analyses showed that HDAC9 levels were markedly enhanced in the ischemic cerebral hemisphere (ischaemic core and penumbra). To further determine which kinds of cells express HDAC9 in the brain, confocal immunofluorescent analysis was performed in this study. We identified neurons by staining for the neuronal nuclear marker NeuN, astrocytes by staining for GFAP, activated microglia/macrophages by staining for CD11b and BMVECs by staining for CD34. It was found that HDAC9 was expressed in all these cells with a relative low expression in microglia (Fig. 1C), which was further confirmed by RT‐PCR (Fig. 1D) and Western blot (Fig. 1E) analyses in primary cultured cortical neurons, astrocytes, microglia and BMVECs.

### Gene silencing of HDAC9 ameliorated cerebral injury in ischaemic stroke

**Figure 2:**
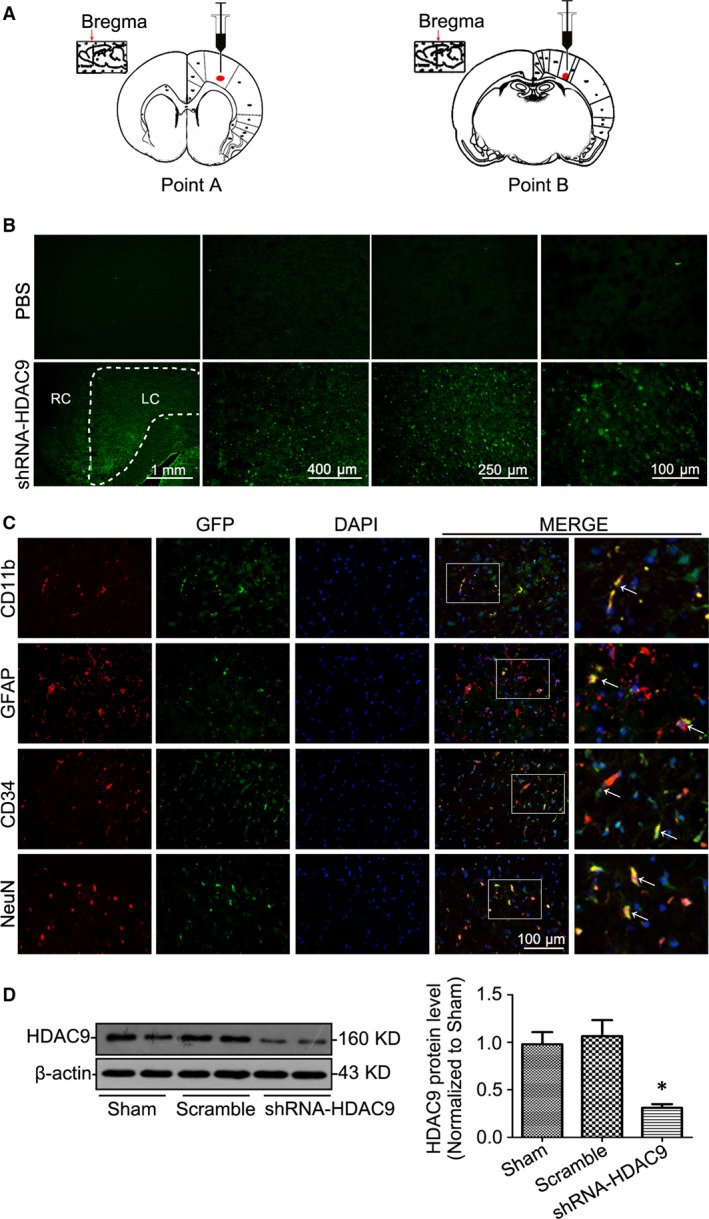
In vivo gene silencing of HDAC9 by intracortex lentiviral gene delivery in rats. (**A**) Representative figures showing the position of intracortex injection *via* using a stereotaxic instrument. (**B**) Representative cortex fluorescent photomicrographs of the brain 1 week after intracortex delivery of lentivirus pGLV3‐shRNA‐HDAC9 encoding green fluorescent protein (GFP) demonstrating predominant localization of GFP expression in the left cortex of rats. Injection of PBS was used as a negative control. LC: left cortex; RC: right cortex. (**C**) Representative confocal microscopic images showing GFP expression in cerebral cells including neuron, microglia, astrocytes and BMVECs 1 week after stereotaxic injection of lentivirus pGLV3‐shRNA‐HDAC9 through confocal immunofluorescent analysis using specific anti‐GFP monoclonal rabbit antibody and antibodies for different cell type markers. (**D**) Representative Western blot gel documents and summarized data showing the efficiency of gene silencing of HDAC9. *P < 0.05 *versus* sham‐operated rats (*n* = 8).

To address the role of HDAC9 in ischaemic stroke *in vivo*, we infused pGLV3‐shRNA‐HDAC9 into the left cerebral cortex of rats by stereotaxic injection as shown in Figure 2A. After 1 week injection, intense green fluorescence was seen in the cerebral cortex after delivery of the lentivirus pGLV3‐shRNA‐HDAC9 encoding GFP, but not in control brains in rats injected with PBS. In addition, we found the predominant localization of GFP expression in the left cortical areas (Fig. 2B). We further examined which kind of cells gene silencing occurred as demonstrated by GFP expression after stereotaxic injection of lentivirus through confocal immunofluorescent analysis using specific anti‐GFP monoclonal rabbit antibody and antibodies for different cell type markers. Our results showed that GFP was expressed in different cerebral cells including neuron, microglia, astrocytes and BMVEC (Fig. 2C). The efficiency of HDAC9 gene silencing in the left cerebral cortex of rats was further confirmed by Western blot analysis (Fig. 2D).

**Figure 3:**
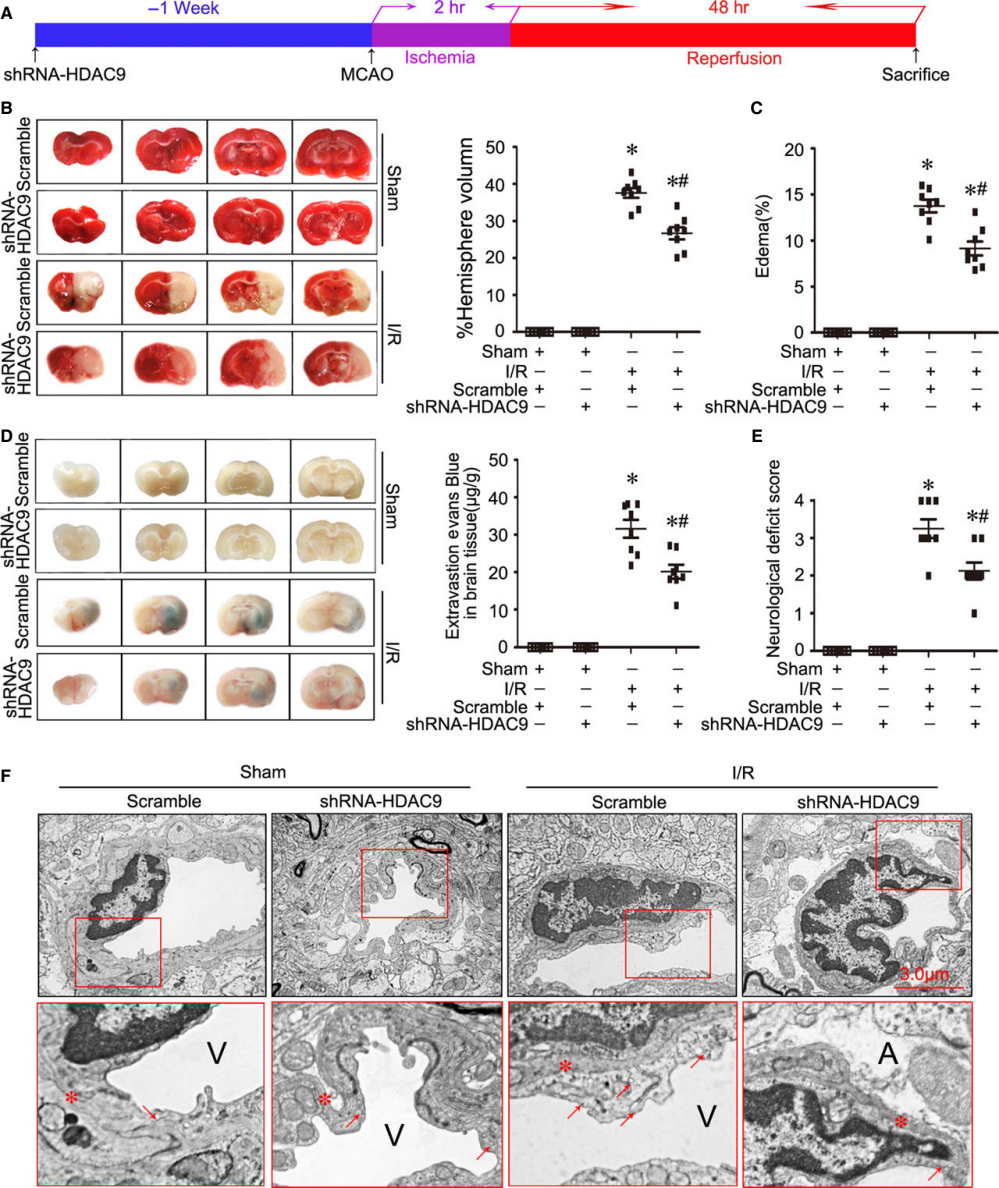
Gene silencing of HDAC9 ameliorated cerebral injury in ischemic stroke. (**A**) A representative figure showing the timeline of surgery in this study. (**B**) Representative photographs of 2,3,5‐triphenyltetrazolium chloride (TTC) staining (left panel) and calculated infarct volume in ischemic rats at 48 hrs of reperfusion after 2 hrs middle cerebral artery occlusion (MCAO). (**C**) Calculated cerebral edema in ischaemic rats at 48 hrs of reperfusion after MCAO. (**D**) Representative microscopic images of brain sections after injection of Evans blue (left panel) and measured Evans blue intensity in ischaemic rats at 48 hrs of reperfusion after MCAO. (**E**) Neurologic deficit scores in ischemic rats at 48 hrs of reperfusion after MCAO. (**F**) Transmission electronic microscope analysis of the blood–brain barrier (BBB) integrity showing that in ischaemic rats, the endothelial cells and their nucleus were swollen and deformed, and the integrity of BBB was destroyed, presenting perivascular edema, vacuolation (red arrows indicated) and membrane (red stars indicated) damage. A represents astrocytes; V represents blood vessel; *n* = 8 animals per group. *P < 0.05 *versus* sham‐operated rats; ^#^ *P* < 0.05 *versus* scramble I/R rats.

We then induced ischaemia in the left hemisphere by MCAO and tested neurological outcome and infarct size at 48 hrs after reperfusion, the timeline of surgery was summarized in Figure 3A. By TTC staining and Evans blue permeability assays, we observed that gene silencing of HDAC9 reduced infarction volume (Fig. 3B), edema formation (Fig. 3C) and BBB permeability dysfunction (Fig. 3D), as well as neurological deficit in ischaemic rats (Fig. 3E). Furthermore, electron microscopy analysis showed that in ischemic rats, the endothelial cells and their nucleus were swollen and deformed, and the integrity of BBB was destroyed, presenting perivascular edema, vacuolation and membrane damage (Fig. 3F). However, gene silencing of HDAC9 ameliorated ischaemia‐induced endothelial injury and BBB disruption.

### Gene silencing of HDAC9 reduced inflammatory responses and apoptosis in BMVEC under OGD

**Figure 4:**
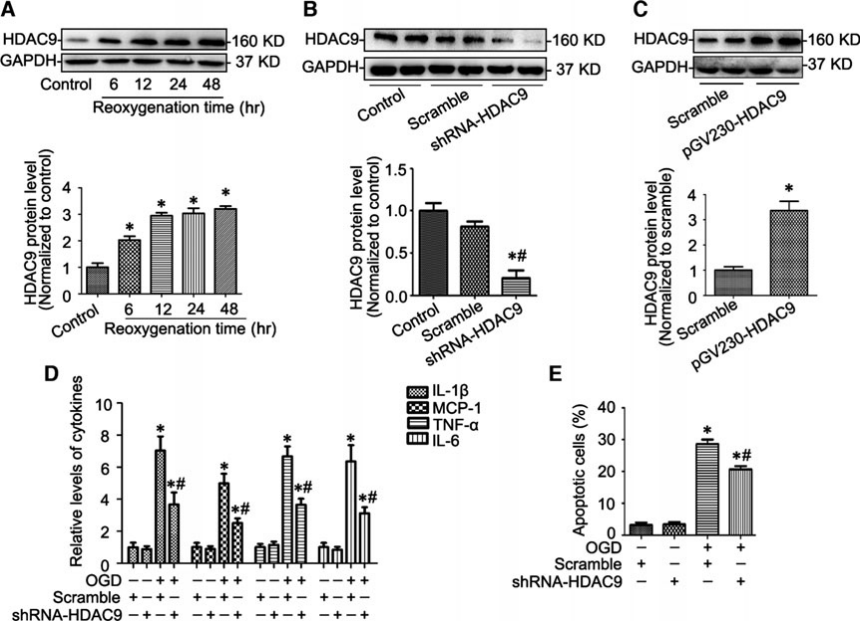
Gene silencing of HDAC9 reduced inflammatory responses and apoptosis in brain microvessel endothelial cells (BMVECs) under OGD (**A**) Representative Western blot gel documents and summarized data showing the HDAC9 protein levels in BMVECs cultured by the model of OGD (1‐hr OGD followed by different time‐points of reoxygenation including 6, 12, 24 or 48‐hr). (**B**) Representative Western blot gel documents and summarized data showing the efficiency of gene silencing of HDAC9 after 24‐hr shRNA‐HDAC9 transfection. (**C**) Representative Western blot gel documents and summarized data showing the efficiency of HDAC9 overexpression after 24‐hr pGV230‐HDAC9 transfection. (**D**) The levels of pro‐inflammatory mediators in BMVECs cultured by the model of OGD (1‐hr OGD followed by 24‐hr reoxygenation) with or without gene silencing of HDAC9. (**E**) Summarized data showing cell apoptosis determined by flow cytometric analysis in BMVECs with different treatments. *P < 0.05 *versus* scramble; ^#^ *P* < 0.05 *versus* BMVECs cultured by the model of OGD (*n* = 6).

As shown in Figure 4A, OGD significantly induced HDAC9 expression in BMVECs in a time‐dependent manner. To further investigate the role of HDAC9 on BMVEC function, gene silencing of HDAC9 by shRNA‐HDAC9 (Fig. 4B) or overexpression of HDAC9 by pGV230‐HDAC9 transfection (Fig. 4C) was used in this study. We found that OGD‐induced the production of pro‐inflammatory mediators was attenuated by HDAC9 knockdown (Fig. 4D), as well as the apoptosis measured *via* the flow cytometry (Fig. 4E).

### HDAC9 contributed to OGD‐induced abnormal endothelial cell permeability

**Figure 5:**
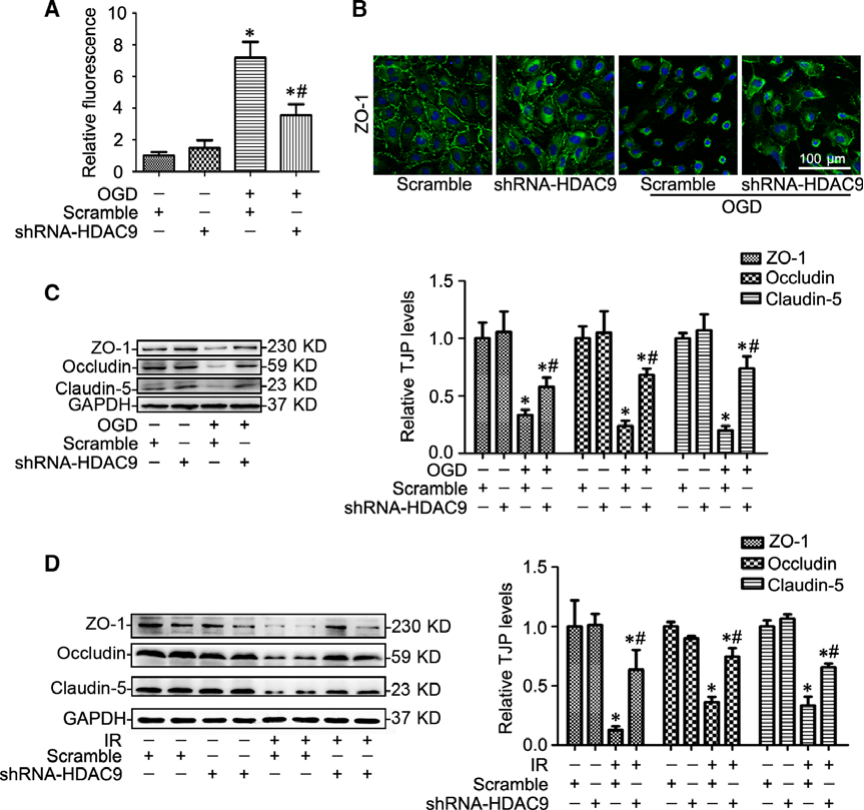
HDAC9 contributed to OGD‐induced abnormal endothelial cell permeability. (**A**) Summarized data showing the BMVECs permeability changes in BMVECs cultured by the model of OGD (1‐hr OGD followed by 24‐hr reoxygenation) with or without gene silencing of HDAC9. (**B**) Representative images showing that ZO‐1 protein changes in BMVECs with different treatments. (**C**) Representative Western blot gel documents and summarized data showing tight‐junction protein (TJP) levels in BMVECs with different treatments. *P < 0.05 *versus* scramble; ^#^ *P* < 0.05 *versus* BMVECs cultured by the model of OGD (*n* = 6). (**D**) Representative Western blot gel documents and summarized data showing TJP levels in ischaemic rats at 48 hrs of reperfusion after 2‐hr MCAO. *P < 0.05 *versus* sham‐operated rats; ^#^ *P* < 0.05 *versus* scramble I/R rats (*n* = 8).

Furthermore, we found that gene silencing of HDAC9 ameliorated the OGD‐induced abnormal endothelial cell permeability (Fig. 5A). Immunofluorescence analysis clarified the damaged ZO‐1 protein was recovered after gene silencing of HDAC9 (Fig. 5B), accompanied by increased expression of tight‐junction proteins (TJPs) including ZO‐1, Occludin and Claudin‐5 (Fig. 5C), which was further confirmed by the fact that gene silencing of HDAC9 prevented the decreased expression of TJPs after cerebral I/R injury in rats (Fig. 5D).

### Autophagy was regulated by HDAC9 in BMVECs

**Figure 6:**
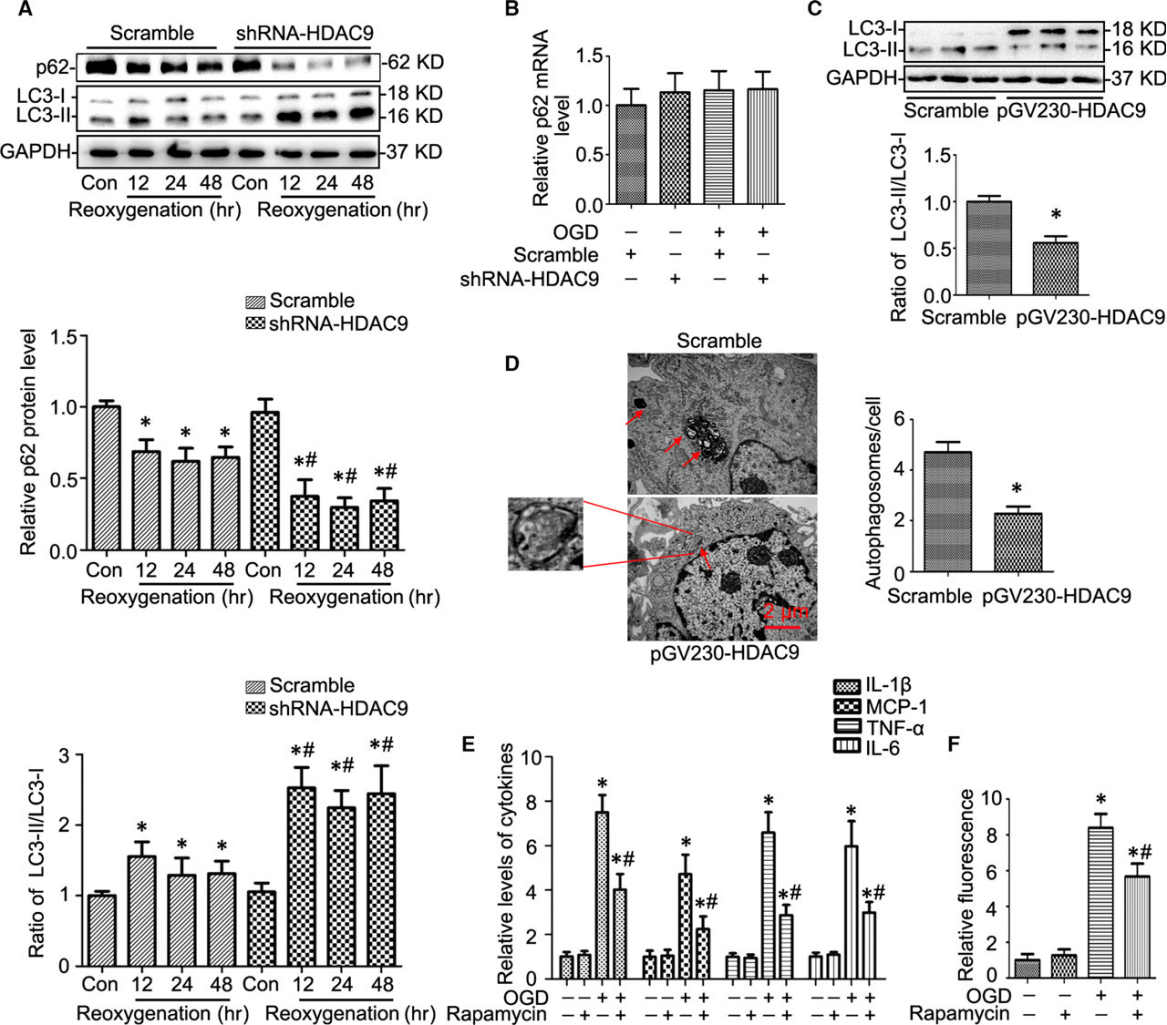
Autophagy was regulated by HDAC9 in BMVECs. (**A**) Western blot gel documents and summarized data showing gene silencing of HDAC9 on the effects of LC3II/I and p62 levels in BMVECs cultured by the model of OGD (1‐hr OGD followed by different time‐points of reoxygenation including 12, 24 or 48‐hr). (**B**) The effect of HDAC9 gene silencing on p62 mRNA levels by real‐time RT‐PCR analysis in BMVECs under OGD condition cultured by the model of OGD (1‐hr OGD followed by 24‐hr reoxygenation). (**C**) Western blot gel documents and summarized data showing the LC3II/I levels in BMVECs after 24‐hr pGV230‐HDAC9 transfection. (**D**) Electronic micrographs showing the typical autophagosomes with double membranes/cell in BMVECs after 24‐hr pGV230‐HDAC9 transfection. (**E**) The levels of pro‐inflammatory mediators in BMVECs treated with rapamycin under OGD condition (1‐hr OGD followed by 24‐hr reoxygenation). (**F**) Summarized data showing the BMVECs permeability changes in BMVECs treated with rapamycin under OGD condition (1‐hr OGD followed by 24‐hr reoxygenation). *P < 0.05 *versus* scramble; ^#^ *P* < 0.05 *versus* BMVECs cultured by the model of OGD (*n* = 6).

A growing body of studies have suggested that loss of autophagy contributes to endothelial dysfunction in vascular biology and autophagy is connected to the expression of inflammatory cytokines and the tight junction proteins. Therefore, we examined whether HDAC9 regulates autophagy in BMVECs. As shown in Figure 6A, gene silencing of HDAC9 increased LC3II/I ratio and reduced p62 levels in BMVECs under OGD. We also examined p62 mRNA levels and found that gene silencing of HDAC9 had no effect on p62 mRNA expression under OGD condition indicating that the decrease in p62 protein level is because of its degradation alongside polyubiquitinated proteins destined for autophagosomes rather than decreased transcription (Fig. 6B). To further clarify the role of HDAC9 on the regulation of autophagy, overexpression of HDAC9 was used in this study. We found that overexpression of HDAC9 decreased the LC3II/I ratio (Fig. 6C) and the number of typical autophagosomes with double membranes examined by transmission electron microscopy (Fig. 6D). Collectively, these results indicated that HDAC9 is associated with the regulation of autophagy. To examine the role of HDAC9‐suppressed autophagy in BMVECs, we used the autophagy enhancer rapamycin to restore autophagy as control. Our study showed that OGD induced inflammatory response (Fig. 6E) and endothelial cell permeability dysfunction (Fig. 6F), which were alleviated by restoring autophagy with low dose of rapamycin as well as by gene silencing of HDAC9 as indicated before.

## Discussion

Although recent studies have highlighted the importance of HDAC‐mediated epigenetic processes in the development of ischemic stroke, the molecular events to induce cerebral injury are not fully understood. In this study, we found that HDAC9 was up‐regulated in the ischaemic cerebral hemisphere after cerebral I/R injury in rats and *in vivo* gene silencing of HDAC9 by recombinated lentivirus infection and stereotaxic injection in the brain reduced cerebral injury in experimental stroke. We further demonstrated that endothelial injury was associated with exacerbating inflammation and suppressing autophagy induced by HDAC9. This study for the first time provides direct evidence that HDAC9 is one of the critical components of a signal transduction pathway that links cerebral injury to epigenetic modification in the brain.

Dysfunction of the vascular endothelium plays a central role in the pathogenesis of many human diseases such as cardiovascular disease, stroke, diabetes and chronic kidney failure 22. Brain endothelial cells have unique properties in terms of barrier function, regulation of local CBF and interactions with other members of the neurovascular unit 23. In particular, brain endothelial cells is the structural basis of the BBB, which serves an important role of restricting the entry of molecules and immune cells from the systemic circulation into the CNS 1. We found that except that HDAC9 contributed to OGD‐induced inflammation, endothelial cell permeability and apoptosis, HDAC9 also reduced autophagy. In the CNS, autophagy is triggered as an adaptive mechanism by various stressors, including cerebral ischaemia, nutrient deprivation or excitotoxic stimuli, providing nutrients and eliminating damaged organelles to promote cell survival 24. Studies have revealed the accumulation of autophagy within neurons and astrocytes in experimental models of cerebral ischaemia both *in vivo* and *in vitro*. Some of them indicate that the autophagy‐induced neuroprotection effect is essential for neurons survival 25, 26, but others also report that autophagy can cause autophagic neurons death aggravating the ischaemia outcome 27, 28, 29. The mixed observation may result from the possible different contribution of autophagy in different diseased states or different experimental animal models used. With respect to autophagy in endothelial cells, a growing body of studies have suggested that loss of autophagy may be a central mechanism through which risk factors elicit endothelial dysfunction in vascular biology 30. In consistent with a very recent study showing that rapamycin induces protective autophagy in vascular endothelial cells exposed to OGD 31, we found that OGD‐induced endothelial cell inflammatory responses and permeability dysfunction were alleviated by rapamycin. Taken together, these results provide direct evidence that HDAC9 plays an important role in autophagy and endothelial function, indicating that basal autophagy is essential in maintaining the architectural integrity of BBB and the inhibition of autophagy by HDAC9 may have severe negative impact on endothelial functions. In fact, an increasing number of studies support the link between autophagy and inflammation. Down‐regulation of autophagy enhances the inflammatory response and thus represents a possible common pathogenic event underlying a number of autoinflammatory syndromes, such as tumour necrosis factor receptor‐associated periodic syndrome 32. In addition, studies have demonstrated that autophagy enhances intestinal epithelial tight junction barrier function by targeting claudin‐2 protein degradation 33. Although in this study, we found that HDAC9 contributed to OGD‐induced BMVEC dysfunction by the increased inflammatory responses, cellular apoptosis and endothelial cell permeability and suppressed autophagy, we did not explore how autophagy is connected to the expression of inflammatory cytokines and the tight junction proteins in this study. Also, we cannot exclude that other mechanisms, which are independent or crosstalk with autophagy, may be involved in this process. In addition, studies have demonstrated that acetylation can regulate autophagy 34 and increased cellular acetylation levels by HDAC inhibition in cells promote autophagy 35. A very recent study from our group has also demonstrated that HDAC4 contributes to podocyte injury by inhibition of autophagy in diabetic nephropathy 36. Therefore, it is necessary to further explore the mechanisms by which HDAC9 mediates autophagy.

It should be noted that although we focus on the role of HDAC9 in BMVECs, we cannot exclude the effect of HDAC9 on the regulation of other cell types in ischemic brain. In this study, we also found that OGD could induce HDAC9 expression in primary cultured neuron, microglia and astrocytes (Fig. S1). In particular, ischaemia can cause a series of pathophysiological changes of neurons such as trigger axonal sprouting, dendrite outgrowth, spine morphogenesis and even induce neuron death 37, 38. Emerging evidence has indicated that HDAC9 is linked to neuronal physiology and pathology 39. Sugo *et al*. have reported that HDAC9 regulates gene expression and dendritic growth in developing cortical neurons 40. Regarding of the context of neuronal pathology, HDAC9 has been reported to be associated with schizophrenia 41. Lang *et al*. reported that HDAC9 is hemizygously deleted in a small proportion of schizophrenia patients. They also observed that HDAC9 is widely expressed in areas of the mouse brain associated with the neuropathology of schizophrenia, and the expression is exclusively detected in post‐mitotic neurons, indicating that HDAC9 is of importance for the correct function of mature neurons 41. Therefore, it is necessary to further clarify the role of HDAC9 on the regulation of neuronal function and whether autophagy in neurons shares the same mechanisms as BMVECs regulated by HDAC9 in ischaemic stroke.

In conclusion, this study indicates that HDAC9 contributes to endothelial cell injury and demonstrates that HDAC9 is one of critical components of a signal transduction pathway that links ischemic cerebral injury to reduced autophagy in experimental stroke. Pharmacological targeting of HDAC9‐mediated signalling pathways at multiple levels may help design a new approach to develop therapeutic strategies for prevention of deterioration of cerebral function and for the treatment of stroke.

## Conflicts of interest

The authors confirm that there are no conflicts of interest.

## Supporting information


**Figure S1** Western blot gel documents and summarized data showing the HDAC9 protein levels in different cells including primary cortical neuron (**A**), microglia (**B**) and astrocytes (**C**) under OGD condition. *P < 0.05 *versus* control (*n* = 6).
**Table S1** Primer pairs of target genes used for real time RT‐PCR in this study.
**Table S2** Antibodies used in this study.
