# Supplementary material for: HDAC9 exacerbates endothelial injury in cerebral ischaemia/reperfusion injury
Source: J Cell Mol Med. 2016 Feb 10;20(6):1139–49. doi: 10.1111/jcmm.12803 (PMC4882992; doi:10.1111/jcmm.12803)
Supplement: Supplementary file 1 — Figure S1 Western blot gel documents and summarized data showing the HDAC9 protein levels in different cells including primary cortical neuron (A), microglia (B) and astrocytes (C) under OGD condition. *P < 0.05 versus control (n = 6). Table S1 Primer pairs of target genes used for real time RT‐PCR in this study. Table S2 Antibodies used in this study. [file JCMM-20-1139-s001.doc]

**SUPPORTING INFORMATION**

**HDAC9** **exacerbates endothelial injury in cerebral ischemia/reperfusion injury**

Weichen Shi^1,2^, Xinbing Wei^1^, Ziying Wang^1^, Huirong Han^1^, Yi Fu^1^, Jiang Liu^1^, Yan Zhang^1^, Jian Guo^1^, Chuanqiao Dong^1^, Di Zhou^1^, Quan Zhou^1^,Yuxin Chen^2^, and Fan Yi^1*^

^1^Department of Pharmacology, Shandong University School of Medicine, Jinan, China, 250012

^2^Department of Hepatobiliary Surgery, Qilu Hospital of Shandong University, Jinan, China, 250012,

Running title: HDAC9 and ischemic stroke

*

Send Correspondence and

Reprint Requests to: Fan Yi

Department of Pharmacology

Shandong University School of Medicine

44#, Wenhua Xi Road,

Jinan, Shandong, 250012, P.R. China

Phone : 86-0531-88382616

Fax : 86-0531-88382616

E-mail: [fanyi@sdu.edu.cn](mailto:fanyi@sdu.edu.cn)

**Table S1. Primer pairs of target genes used for real time RT-PCR in this study**

| Genes | Accession No. | Forward | Reverse |
| --- | --- | --- | --- |
| HDAC9 | NM_001200045.1 | GTCCCTGCCCAATATCAC | GCTGTTCGGTTTGCCCTC |
| MCP-1 | NM_031530.1 | CTGGGCCTGTTGTTCACAGTTGC | CTTTGGGACACCTGCTGCTGGTG |
| p62 | [NM_175843.4](http://www.ncbi.nlm.nih.gov/entrez/viewer.fcgi?db=nucleotide&id=402743349) | AGAATGTGGGGGAGAGCGTGGC | GGGTGTCAGGCGGCTTCTCTT |
| IL-6 | NM_012589.2 | CCGGAGAGGAGACTTCACAGAG | CAGTGCATCATCGCTGTTCATAC |
| TNFα | XM_008772775.1 | CCACCACGCTCTTCTGTCTA | TTTGCTACGACGTGGGCTAC |
| IL-1β | NM_031512.2 | CCAAGCCCTTGACTTGGGCTGTC | TGGGTCCTCATCCTGGAAGCTCC |
| GAPDH | NM_017008.4 | TGCATCCTGCACCACCAACTGC | ACAGCCTTGGCAGCACCAGTGG |

**Table S2. Antibodies used in this study**

| **Primary antibodies** | **Host** | **Dilution and supplier** | **Application** |
| --- | --- | --- | --- |
| HDAC9 | Rabbit | 1:2000(1:100 for IP); abcam, Cambridge, MA | WB, IF, IP |
| LC3B | Rabbit | 1:1000; Cell Signaling, Danvers, MA | WB |
| p62/SQSTM1 | Rabbit | 1:1000; Cell Signaling, Danvers, MA | WB |
| ZO-1 | Rabbit | 1:500 (1:100 for IF); Cell Signaling, Danvers, MA | WB, IF |
| Claudin-5 | Rabbit | 1:1200; ProteinTech Group, Chicago, IL | WB |
| Occludin | Rabbit | 1:1200; ProteinTech Group, Chicago, IL | WB |
| NeuN | Mouse | 1:100; Cell Signaling, Danvers, MA | IF |
| CD11b | Mouse | 1:100; BD Biosciences, San Diego, CA | IF |
| CD34 | Mouse | 1:100; BD Biosciences, San Diego, CA | IF |
| GFAP | Mouse | 1:100; Invitrogen, Carlsbad, CA | IF |
| GFP | Rabbit | 1:20; Santa Cruz,Dallas, Texas,U.S.A | IF |
| GAPDH | Mouse | 1:5000; ProteinTech Group, Chicago, IL | WB |
| β-actin | Mouse | 1:6000; ProteinTech Group, Chicago, IL | WB |


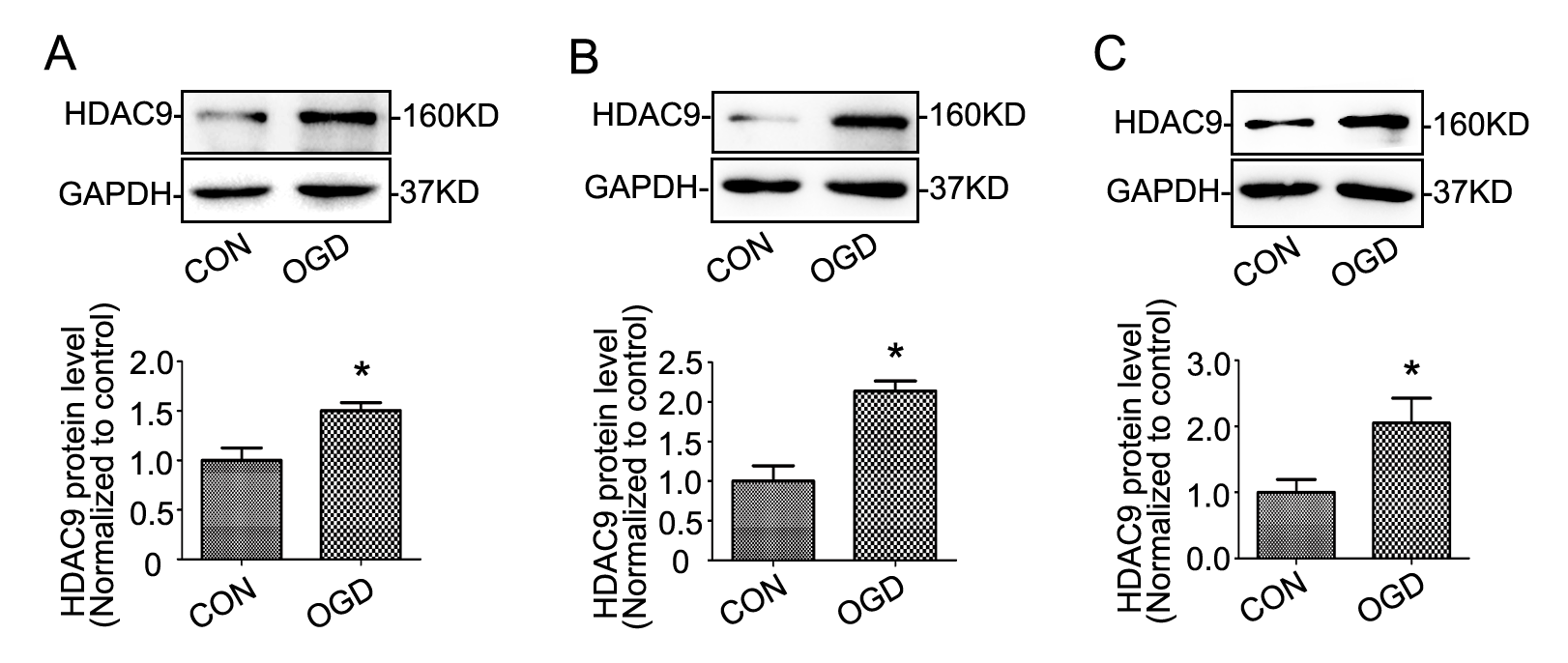


**Fig. S1.** Western blot gel documents and summarized data showing the HDAC9 protein levels in different cells including primary cortical neuron (**A**), microglia (**B**) and astrocytes (C) under OGD condition. * P<0.05 vs. control (n=6).
